# Cardiotoxicity, survival, and dexrazoxane use in patients with soft tissue sarcoma: a Danish population‑based cohort study

**DOI:** 10.2340/1651-226X.2026.45897

**Published:** 2026-07-07

**Authors:** Joanna Vitfell-Rasmussen, Ninna Aggerholm-Pedersen, Christina Brun Knudsen, Kasper Madsen, Lars Fiksen, Niels Junker, Michael Mørk Petersen, Thomas Kümler, Anders Krarup-Hansen, Bodil Elisabeth Engelmann

**Affiliations:** a Department of Oncology, Herlev University Hospital, Herlev, Denmark; b Department of Oncology, Aarhus University Hospital, Aarhus, Denmark; c Musculoskeletal Tumor Section, Department of Orthopedics, Rigshospitalet, University Hospital of Copenhagen, Copenhagen, Denmark; d Department of Cardiology, Herlev University Hospital, Herlev, Denmark; e Department of Clinical Medicine, University of Copenhagen, Copenhagen, Denmark; f Clinical Translational Research Steno Diabetes Center Copenhagen, Herlev, Denmark

**Keywords:** Cardiotoxicity, sarcoma, dexrazoxane, anthracyclines, doxorubicin

## Abstract

**Background and purpose:**

Anthracycline-associated cardiotoxicity is a challenge that dexrazoxane may reduce. The aim of this study was to report the overall survival (OS), incidence of cardiotoxicity and dexrazoxane use in a Danish population-based cohort of patients with soft tissue sarcoma (STS) treated with doxorubicin, olaratumab and dexrazoxane.

**Patients/material and methods:**

We performed a retrospective study of adults (≥18 years) with locally advanced or metastatic STS treated with doxorubicin, olaratumab and dexrazoxane between 2015 and 2019 at Herlev University Hospital (Herlev) and Aarhus University Hospital (Aarhus). Dexrazoxane was administered from cycle 1 at Herlev and from cycle 4 at Aarhus. OS was measured from doxorubicin initiation to death. Cardiotoxicity was defined as a >10 percentage‑point decline in left ventricular ejection fraction to <50% compared with baseline.

**Results:**

In total, 106 patients were included: 71 patients (67%) from Herlev and 35 (33%) from Aarhus. Mean age was 59 years, 59% were female. Median OS was 18 months (95% confidence interval [CI]: 11.0–20.7) and median cumulative doxorubicin dose was 320.7 mg/m² (range: 37.5–605.4). Dexrazoxane was administered to 68 patients (96%) at Herlev and 18 (51%) at Aarhus. Seven patients (7%) developed cardiotoxicity (Herlev *n* = 4; Aarhus *n* = 3). Median OS was 19.6 months at Herlev and 14.8 months at Aarhus (*p* = 0.24). Neutropenic fever occurred in 27 patients (38%) at Herlev and six patients (17%) at Aarhus (*p* = 0.88).

**Interpretation:**

Doxorubicin with olaratumab and dexrazoxane resulted in a low incidence of cardiotoxicity and OS was consistent with previously reported.

## Introduction

The risk of developing anthracycline-associated cardiotoxicity is still a concern when treating patients with doxorubicin.

Soft tissue sarcomas (STS) are a heterogeneous group of solid malignant tumors derived from mesenchymal cells [1]. Treatment is centralized at two specialized sarcoma referral centers in Denmark, both with integrated central pathology. Anthracycline‑based treatment with doxorubicin constitutes the standard first‑line treatment for most patients with advanced or metastatic STS [2].

Cardiotoxicity is an infrequent but potentially detrimental toxicity to anthracycline-based treatment. The precise molecular pathogenesis of this condition is still not entirely understood but it is thought that topoisomerase 2β inhibition by anthracycline can cause double-stranded breaks in DNA, which can lead to cardiomyocyte death [3, 4]. Patient-related cardiotoxicity risk factors include age, radiation therapy, sex, renal failure, infusion rate, preexisting cardiovascular disease and the cumulative doxorubicin dose [5, 6]. For this reason, the number of treatment cycles with doxorubicin is limited to a cumulative dose of 450 mg/m^2^, with 75 mg/m^2^ per treatment cycle to a maximum of six cycles [7].

Olaratumab is a human recombinant monoclonal immunoglobulin G antibody binding to platelet-derived growth factor receptor alpha, which was approved for metastatic STS in combination with doxorubicin after promising results of a phase II trial [8]. In the large randomized, double-blinded phase III ANNOUNCE trial, the additional value of olaratumab to doxorubicin could not be confirmed and for this reason olaratumab was withdrawn in 2019 [9]. The patients enrolled in the ANNOUNCE trial reached a median overall survival (OS) of 20 months. This was a substantial improvement compared to the median OS of 12.8 months reported in an earlier doxorubicin-based first-line treatment trial [10]. Dexrazoxane, a cardio-protectant was administered by the discretion of the investigator as a supportive intervention in both treatment arms, allowing for a maximum of eight doxorubicin cycles and a higher cumulative doxorubicin dose (600 mg/m^2^). Real-world outcomes on the clinical effectiveness of doxorubicin and olaratumab have failed to replicate the OS demonstrated but the use of dexrazoxane was not mentioned [11, 12].

The aim of this study was to report the OS, incidence of cardiotoxicity and dexrazoxane use in a Danish population‑based cohort of patients with advanced or metastatic STS treated with doxorubicin, olaratumab and dexrazoxane.

## Patients/material and methods

### Study population

A retrospective study was conducted between 2015 and 2019 covering all patients with locally advanced or metastatic STS referred for doxorubicin and olaratumab at the Departments of Oncology at Herlev University Hospital (Herlev) and Aarhus University Hospital (Aarhus) in Denmark. Patient were eligible provided they had received doxorubicin and olaratumab with or without dexrazoxane and were ≥18 years.

### Treatment schedule

Patients received olaratumab (20 mg/kg intravenous [iv] in cycle 1 and 15 mg/kg in subsequent cycles) on days 1 and 8 plus doxorubicin (75 mg/m² iv bolus in <30 min) on day 1 of each 21‑day cycle for up to eight cycles.

At Herlev, dexrazoxane (750 mg/m^2^, iv bolus in <30 min) was administered on day 1, cycle 1 prior to doxorubicin and was administered for up to eight cycles. At Aarhus, dexrazoxane (750 mg/m^2^, iv bolus in <30 min) was administered on day 1, cycle 4 prior to doxorubicin and was administered for up to 5 cycles.

Granulocyte colony-stimulating factor (G-CSF) was co-administered with every doxorubicin cycle at Herlev except for patients treated within the ANNOUNCE trial. At Aarhus, G-CSF was administered only by clinical indication.

### Data collection

The clinical information was collected from the patients’ medical records and from the Danish National Sarcoma database [13] and was subsequently stored in a study database (REDCap platform). The database contained comprehensive clinical information including date of referral, comorbidities, histological diagnosis, histological malignancy grade [14], oncological and supportive treatment administered, cardiac function, secondary malignancy, and selected toxicities (neutropenic fever and thrombocytopenia) graded according to Common Terminology Criteria for Adverse Events (CTCAE) version 5.0 [15]. The study was reported in accordance with the STROBE guideline.

### Cardiotoxicity

Cardiac function data were evaluated by multigated acquisition (MUGA) left ventricular function measures or echocardiography, which are in Denmark routinely performed before and approximately every 9 weeks during doxorubicin and at follow-up by clinical indication.

Multiple definitions of cardiac toxicity are used, and as of now no international definition is accepted worldwide [16]. We defined cardiotoxicity as a decline in left ventricular ejection fraction (LVEF) of >10 percentage-point to a LVEF of <50% as compared to baseline [17]. This is in accordance with the European Society of Cardiology (ESC) definition of moderate and asymptomatic cancer treatment-related cardiac dysfunction [5]. An echocardiography had to be performed to confirm the cardiotoxicity diagnosis. Early cardiotoxicity was defined as cardiotoxicity occurring within <1 year and late cardiotoxicity as ≥1 year from initiation of doxorubicin [18]. Recovery of cardiotoxicity was defined as a LVEF of ≥50% after cardiotoxicity had been diagnosed [17].

Cardiovascular comorbidities and risk factors were defined as the presence of established cardiovascular disease and/or major contributors to cardiovascular risk. Cardiac-related medication was defined as pharmacological treatment for any of these conditions.

### Ethics

Data collection was approved by the Capital Region of Denmark, Team for medical records (R-20083740) and approval was also granted by the Danish Data protection Agency (P-2021-51). Individual patient informed consent was not required.

### Statistics

Demographics, diagnostic and follow-up characteristics were summarized as medians and range or interquartile range (IQR) for continuous variables, and number and percentages for categorical variables. Survival curves were constructed by Kaplan–Meier´s method. Pairwise comparisons for OS and cardiotoxicity/cumulative dose were performed using a Log Rank test (Mantel-Cox). Group comparisons were performed with Wilcoxon rank-sum test for continuous variables and Fisher’s exact test for categorical variables. OS was defined as the time from doxorubicin initiation to death from any cause and was reported as the median with corresponding 95% confidence intervals (CI).

A landmark analysis was conducted to compare survival according to cumulative doxorubicin dose levels. OS was defined as survival beyond 300 days from the initiation of doxorubicin, and patients were grouped according to the cumulative doxorubicin dose received at 300 days. A separate landmark analysis was conducted to compare survival according to cardiotoxicity. OS was defined as survival beyond 1 year from the initiation of doxorubicin, and patients were grouped based on the occurrence of a cardiotoxic event within the first year.

Cumulative incidence function with death as a competing risk was conducted to estimate the incidence of cardiotoxicity. Time to cardiotoxicity was defined as the interval from initiation of doxorubicin to the clinical diagnosis of cardiotoxicity.

*P* < 0.05 was regarded as statistically significant. To account for multiple comparisons in Table 1, *p*-values were adjusted using the Holm-Bonferroni correction. All statistical calculations were performed using the R software package version 4.3.0.

**Table 1:** Patients’ characteristics.

	Value (*N* = 106)			
	Herlev (*N* = 71)	Aarhus(*N* = 35)	*P*-values (adjusted *P*-value)	Total
Age (years, median, range)	61 (49–70)	58 (45–67)	0.203 (1)	59 (47–69.2)
Body mass index (median, range)	25.8 (23.5–29.9)	25.4 (23.6–27.7)	0.634 (1)	25.4 (23.6–28.8)
Sex (*n*, %) Female Male	42 (59%) 29 (41%)	21 (60%) 14 (40%)	1 (1)	63 (59%) 43 (41%)
Histology (*n*, %) Liposarcoma UPS* Leiomyosarcoma Uterine leiomyosarcoma Other	15 (21%) 6 (9%) 2 (3%) 11 (15%) 37 (52%)	7 (20%) 5 (14%) 6 (17%) 2 (6%) 15 (43%)	0.062 (1)	22 (21%) 11 (10%) 8 (8%) 13 (12%) 52 (49%)
Site of primary tumor (*n*, %) Retroperitoneal Extremities Superficial trunk Uterine Other	13 (18%) 14 (20%) 10 (14%) 16 (23%) 18 (25%)	7 (20%) 8 (23%) 1 (3%) 3 (8%) 16 (46%)	0.074 (1)	20 (18%) 22 (22%) 11 (10%) 19 (18%) 34 (32%)
Tumor grade (*n*, %) ** 1 2 3	11 (16%) 12 (17%) 47 (67%)	2 (6%) 11 (31%) 22 (63%)	0.130 (1)	13 (12%) 23 (22%) 69 (66%)
Cardiovascular comorbidities and risk factors (*n*, %) ** Yes No	34 (49%) 36 (51%)	14 (40%) 21 (60%)	0.533 (1)	48 (46%) 57 (54%)
Cardiac medication, comorbidity yes (*n*, %) Yes No	33 (97%) 1 (3%)	13 (93%) 1 (7%)	0.503 (1)	46 (96%) 2 (4%)
Performance status (*n*, %) *** 0 1 2	63 (91%) 5 (7%) 1 (2%)	30 (86%) 5 (14%) 0 (0%)	0.537 (1)	93 (89%) 10 (10%) 1 (1%)
Cumulative doxorubicin dose mg/m^2^ (median, range)	320 (37.5–605.4)	321.4 (71–600)	0.798 (1)	320.7 (37.5–605.4)
Median number of doxorubicin cycles (median, range)	5 (1–12)	5 (1–8)	0.798 (1)	5 (1–12)
Cumulative doxorubicin dose mg/m^2^ (*n*, %) <450 mg/m^2^ ≥450 – <600 mg/m^2^ ≥600 mg/m^2^	45 (64%) 23 (32%) 3 (4%)	20 (57%) 12 (34%) 3 (9%)	0.617 (1)	65 (61%) 35 (33%) 6 (6%)
Doxorubicin dose reduction (*n*, %) Yes No	39 (55%) 32 (45%)	16 (46%) 19 (54%)	0.413 (1)	55 (52%) 51 (48%)
Dexrazoxane (*n*, %) Yes No	68 (96%) 3 (4%)	18 (51%) 17 (49%)	<0.001 (<0.001)	86 (81%) 20 (19%)
G-CSF (*n*, %) **** Yes No	58 (82%) 13 (18%)	10 (29%) 25 (71%)	<0.001 (<0.001)	68 (64%) 38 (36%)
Continuation of olaratumab (*n*, %) Yes No	21 (30%) 50 (70%)	12 (34%) 23 (66%)	0.660 (1)	33 (31%) 73 (69%)
Median number of olaratumab cycle (median, range)	4 (1–27)	4 (1–34)	0.743 (1)	4 (1–34)
Trabectedin (*n*, %) Yes No	48 (68%) 23 (32%)	21 (60%) 14 (40%)	0.517 (1)	69 (65%) 37 (35%)
Neutropenic fever (*n*, %) ***** Yes No	27 (38%) 44 (62%)	6 (17%) 29 (83%)	0.044 (0.880)	33 (31%) 73 (69%)
Tobacco (*n*, %) *** Yes No	26 (38%) 43 (62%)	9 (26%) 26 (74%)	0.275 (1)	35 (34%) 69 (66%)
Number of lines of treatment (median, range)	4 (1–10)	3 (1–7)	0.030 (0.630)	3 (1–10)
LVEF at baseline (median, IQR) ******	68% (63–75)	64% (57.8–69.2)	0.006 (0.132)	66% (61.2–73)
Best imaging response (*n*, %) ******* Partial response Stable disease Progressive disease	10 (14%) 34 (48%) 27 (38%)	1 (3%) 23 (70%) 9 (27%)	0.080 (1)	11 (11%) 57 (55%) 36 (34%)
Cardiotoxicity (*n*, %) Yes No	4 (6%) 67 (94%)	3 (9%) 32 (91%)	0.682 (1)	7 (7%) 99 (93%)

* Undifferentiated pleomorphic sarcoma.

** One missing value for patients at Herlev.

*** Two missing values for patients at Herlev.

**** Granulocyte colony-stimulating factor.

***** Only accounted for during treatment with doxorubicin.

****** Interquartile range.

******* Two missing values for patient at Aarhus.

## Results

### Total study population

A total of 106 patients were included, 71 patients (67%) at Herlev and 35 patients (33%) at Aarhus (see Table 1). The mean age was 59 years (range: 47–69.2) with a female predominance of 59%. The most common histological subtype was liposarcoma, detected in 22 patients (21%). A total of 69 patients (66%) had grade 3 tumors. A performance status (PS) of 0 was observed in 93 patients (89%). Pre-existing cardiovascular comorbidities and risk factors were present in 48 patients (46%) of whom 96% received cardiac-related medication. Hypertension was most common (*n* = 34), followed by hypercholesterolemia (*n* = 13), diabetes (*n* = 6), atrial fibrillation (*n* = 5), cerebrovascular disease (*n* = 4), valvular heart disease (*n* = 4), thrombosis (*n* = 3), ischemic heart disease (*n* = 2) and other cardiac arrhythmias (*n* = 1). Among these, six patients (6%) were classified as high risk for cardiovascular toxicity [5].

**Figure 1:**
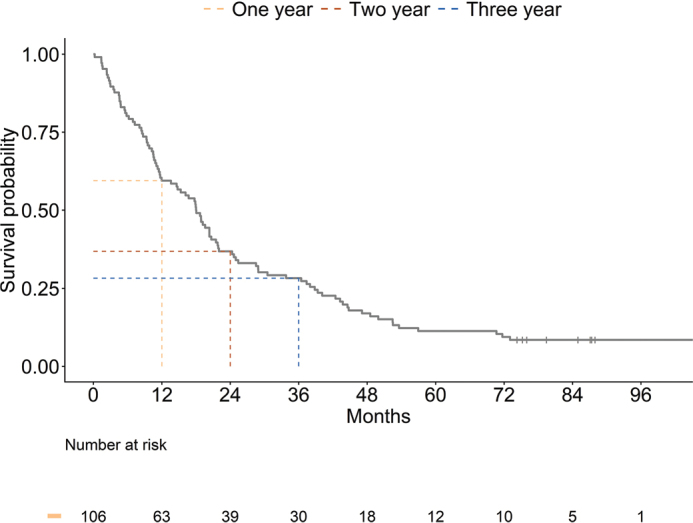
Overall survival of the entire cohort.

Median OS was 18 months (95% CI: 11.0–20.7). At 1 year follow-up 59% of patients were alive (95% CI: 49.5–68.1); at 2 years 37% were alive (95% CI: 27.7–45.9); and at 3 years 28% were alive (95% CI: 20.1–37.1) (see Figure 1). Median length of follow-up was 85 months (IQR: 76.0–87.4). Median number of doxorubicin cycles was five (range: 1–12) with a median cumulative doxorubicin dose of 320.7 mg/m^2^ (range: 37.5–605.4). A total of 55 patients (52%) experienced a doxorubicin dose reduction and main cause was neutropenic fever in 16 patients (30%). Median LVEF at baseline was 66% (IQR: 61.2–73).

**Figure 2:**
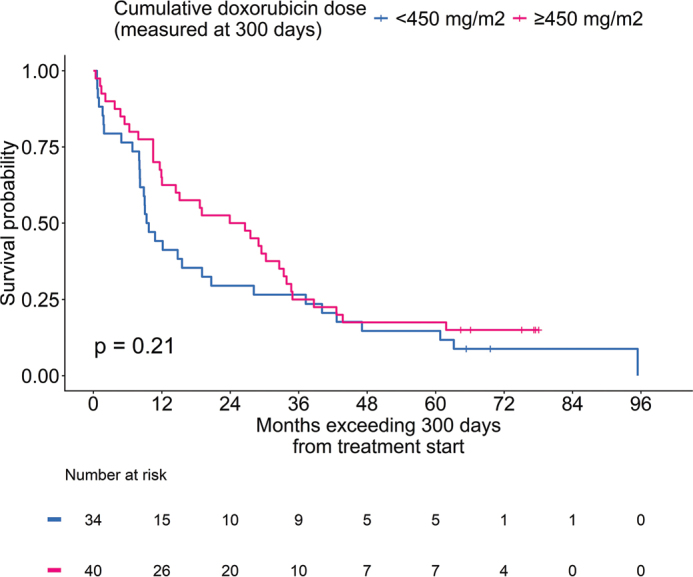
Overall survival according to cumulative doxorubicin dose (*One patient did not receive the total cumulative doxorubicin dose within 300 days as the patient was rechallenged with doxorubicin at a later time point*).

In addition, a total of 65 patients received a doxorubicin cumulative dose of <450 mg/m^2^ (range: 37.5–428.6) of which 71% received dexrazoxane, 35 patients received a doxorubicin cumulative dose ≥450 – <600 mg/m^2^ (range: 450–597.7) of which 97% received dexrazoxane, and six patients received a doxorubicin cumulative dose of ≥600 mg/m^2^ (range: 600–605.4) of which a 100% received dexrazoxane. The six patients were grouped together with those who received a cumulative doxorubicin dose ≥450 – <600 mg/m². Dose rounding explains the doxorubicin cumulative dose of ≥600 mg/m^2^. There was no statistically significant difference in OS and cumulative doxorubicin dose (*p* = 0.21) (see Figure 2).

Olaratumab was continued in 33 patients (31%) after doxorubicin was completed. Median number of cycles was 4 (range: 1–34). A total of 69 patients (65%) received trabectedin and 35 patients (33%) received pazopanib. Trabectedin was administered as a second-line treatment in 37 patients.

Serial MUGA assessments were performed in 85 patients (80%) who were evaluable for cardiotoxicity. For baseline assessment, a single MUGA scan was performed in 20 patients, with no further cardiac imaging conducted. In one patient no cardiac imaging was performed. The main cause for missing cardiac imaging was progressive disease or poor PS, accounting for 62% of patients. Median cumulative doxorubicin dose was 138.36 mg/m^2^ (range: 58.54–375) in this group.

### Outcomes according to treatment center

**Figure 3:**
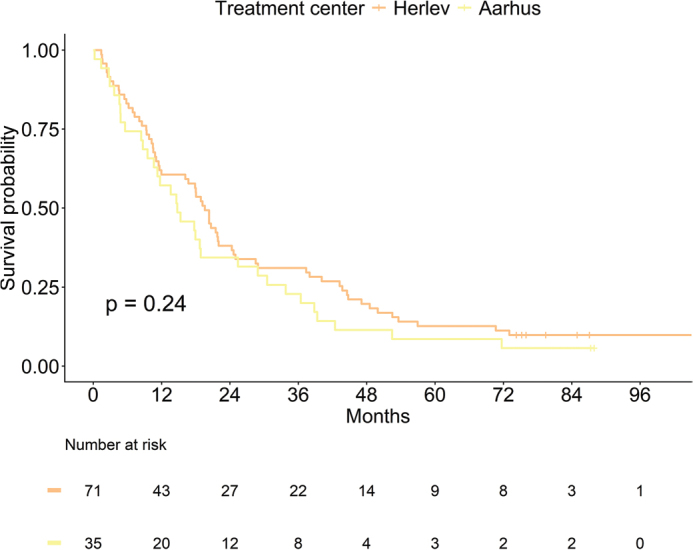
Overall survival according to treatment center.

Median OS was 19.6 months (95% CI: 11.6–22.0) at Herlev, where dexrazoxane was administered from cycle 1 and 14.8 months (95% CI: 9.5–18.8) at Aarhus, where dexrazoxane was administered from cycle 4 (*p* = 0.24) (see Figure 3).

In addition, median number of doxorubicin cycles was five at Herlev (range: 1–12) and five at Aarhus (range: 1–8) with a median cumulative doxorubicin dose of 320 mg/m^2^ (range: 37.5–605.4) at Herlev and 321.4 mg/m^2^ (range: 71–600) at Aarhus. Median LVEF at baseline was 68% (IQR: 63–75) at Herlev and 64% (IQR: 57.8–69.2) at Aarhus (*p* = 0.13).

Dexrazoxane was administered to 68 patients (96%) at Herlev and to 18 patients (51%) at Aarhus (*p* < 0.001). Neutropenic fever was observed in 27 patients (38%) at Herlev and in six patients (17%) at Aarhus (*p* = 0.88). Doxorubicin dose reductions were reported in 39 patients (55%) at Herlev and 16 patients (46%) at Aarhus. G-CSF was administered to 58 patients (82%) at Herlev and 10 patients (29%) at Aarhus (*p* < 0.001).

### Cardiotoxicity

Seven patients (7%) developed cardiotoxicity: four patients (6%) at Herlev and three patients (9%) at Aarhus. Early cardiotoxicity (86%) was more common than late (see Table 2). The 1-year cumulative incidence of cardiotoxicity in the entire cohort was 6% (95% CI: 2–11). Median time to development of cardiotoxicity, among patients who developed cardiotoxicity, was 4.3 months (range: 1.9–19.3).

**Table 2:** Cardiotoxicity characteristics.

Characteristics	Value (*N* = 7)
Type of cardiotoxicity (*n*, %) Early	6 (86%)
Symptoms at diagnosis (*n*, %) Yes No	4 (57%) 3 (43%)
Reason for cardiotoxicity (*n*, %) Doxorubicin Doxorubicin plus trabectedin*	4 (57%) 3 (43%)
Referral to cardiologist (*n*, %) Yes	7 (100%)
Follow-up at cardiologist (*n*, %) Yes	6 (86%)
Initiation of anti-congestive medication (*n*, %) Yes	6 (86%)
Cardiac biomarkers at diagnosis (*n*, %) ** No	6 (86%)
LVEF level at cardiotoxicity (median, range)	42% (40–49)
LVEF during follow-up (*n*, %) LVEF increase to ≥ 50 LVEF increase to ≥ 50 but drops again to <50 LVEF remains < 50	3 (42%) 2 (29%) 2 (29%)
Dexrazoxane before cardiotoxicity (*n*, %) Yes No	5 (71%) 2 (29%)
Cardiovascular comorbidities and risk factors (*n*, %) Yes No	5 (71%) 2 (29%)

* Administered as single-agents and not as a combination treatment.

** Troponin and creatine kinase-myocardial band.

All patients were referred for a cardiology assessment where cardiotoxicity was confirmed by echocardiography. Median LVEF was 42% (range: 40–49) and median LVEF at confirmatory echocardiography was 40% (range: 25–45). Six patients (86%) commenced anti-congestive medication. At diagnosis, systemic treatment was discontinued in two patients (29%), both of whom were receiving doxorubicin. Treatment was paused in two patients (29%), both receiving trabectedin. In the remaining three patients (42%), observation or systemic treatment was continued. Single-agent doxorubicin was considered the main cause for cardiotoxicity in four patients (57%). Single-agent doxorubicin and single-agent trabectedin was considered the main cause in three patients (43%). One patient had received pazopanib for less than 1 week and was therefore not considered a likely cause. At diagnosis, four patients (57%) experienced clinical cardiac-related symptoms including dyspnea and edema.

Median cumulative doxorubicin dose was 374 mg/m^2^ (range: 225–600) in patients with cardiotoxicity and 320 mg/m^2^ (range: 37.5–605.4) in patients without cardiotoxicity (*p* = 0.39). Four patients with cardiotoxicity received a cumulative doxorubicin dose of <450 mg/m^2^ and three patients received a dose of ≥450 mg/m^2^. The incidence of cardiotoxicity was 6% in the <450 mg/m^2^ group and 7% in the ≥450 mg/m^2^ group. Median OS exceeding 1 year in patients with cardiotoxicity was 6.9 months (95% CI: 5.7 – not reached) and 20.1 months (95% CI: 12.3–30.4) in patients without cardiotoxicity (*p* = 0.89).

When excluding the 21 patients (Herlev *n* = 15; Aarhus *n* = 6) with only one MUGA or no cardiac imaging performed, the incidence of cardiotoxicity was 8% in the total study population with 7% at Herlev and 10% at Aarhus (*p* = 0.69).

## Discussion and conclusion

In this retrospective population‑based cohort of patients with advanced or metastatic STS treated with doxorubicin, olaratumab, and dexrazoxane, the median OS was 18 months. This is lower than the 20 month OS reported in the phase III ANNOUNCE trial [9] but higher than the 12.8 months reported in another first-line doxorubicin-based trial [10]. Such differences are expected when comparing real‑world outcomes with results from phase III trials, which typically apply more stringent patient selection criteria.

In the ANNOUNCE trial, the median number of doxorubicin cycles was six with a median cumulative dose of 409 mg/m². Both were higher than in our cohort and may have contributed to the superior OS observed in the ANNOUNCE trial. Median follow‑up duration in the ANNOUNCE trial was 31 months, which is shorter than in our cohort and may also have influenced OS. A PS of 0 was reported in 59% of patients in the ANNOUNCE trial, which is lower than in our cohort and therefore cannot explain the difference in OS. However, when focusing exclusively on patients treated at Herlev, the median OS was 19.6 months which is comparable to the median OS reported in the ANNOUNCE trial.

The incidence of cardiotoxicity was higher in our cohort compared with the 1% reported in the ANNOUNCE trial [19]. This is despite the more frequent use of dexrazoxane in our cohort compared with the 64% of patients receiving dexrazoxane in the ANNOUNCE trial. Of relevance, 46% of patients in our cohort had pre-existing cardiovascular comorbidities and risk factors. However, among high-risk patients, the prevalence was 6%, comparable to 6.5% reported in the ANNOUNCE trial [19]. Nevertheless, pre-existing cardiovascular comorbidities and risk factors may still have contributed to the observed difference [5, 17]. However, directly comparing the incidence of cardiotoxicity between the two studies is challenging because the definitions used are not identical. In the ANNOUNCE trial, cardiac dysfunction was assessed using CTCAE version 4.0, and clinical cardiotoxicity was defined as grade ≥ 3 heart failure according to the same criteria. Using this definition, only four patients (4%) in our cohort met the criteria for clinical cardiotoxicity making the incidence more comparable.

In comparison to other studies, the incidence of cardiotoxicity observed in our cohort was either lower or within similar range. A large retrospective study including 2,671 patients with other cancers reported a 9% incidence of cardiotoxicity without using dexrazoxane [20]. A separate retrospective study from our institution including 75 patients with sarcoma reported a markedly higher 31% incidence of cardiotoxicity, with dexrazoxane administered in only one patient [17]. Another retrospective study from our institution including 454 patients with sarcoma reported a 11% incidence of cardiotoxicity, but a confirmatory echocardiography was not performed and the use of dexrazoxane was not mentioned [21]. Furthermore, a meta-analysis including 49,017 patients with other cancers reported clinical overt cardiotoxicity in 6% of patients, while subclinical cardiotoxicity developed in 18%, the use of dexrazoxane was not mentioned [22]. A recent published retrospective study of 672 patients with sarcoma receiving anthracycline with or without dexrazoxane, using the same cardiotoxicity definition as in our cohort, reported a similar 7.1% incidence of cardiotoxicity [23].

The fact that only a single MUGA or no cardiac imaging was performed in 20% of patients in our cohort may also have contributed to the low incidence of cardiotoxicity observed. Not all patients experience symptomatic cardiotoxicity, and it remains unknown whether some may subsequently have developed cardiotoxicity. When excluding these patients, the incidence of cardiotoxicity was higher in our cohort. Of relevance, the median cumulative doxorubicin dose in this group was low, suggesting a minimal risk of treatment-related cardiotoxicity. Cardiotoxic risk is generally considered to increase at cumulative doxorubicin doses of ≥250 – 300 mg/m² [24]. Since this threshold was not reached for most of the patients mentioned above, we considered it was appropriate to retain them in the cohort. Moreover, our study was not exclusively focused on reporting the incidence of cardiotoxicity but also on evaluating OS, further supporting their inclusion.

Comparison of outcomes according to treatment center revealed no statistically significant differences in OS, incidence of cardiotoxicity, or cumulative doxorubicin dose. Notably, a retrospective study including 672 patients with sarcoma reported that a cumulative doxorubicin dose of >300 mg/m^2^ with dexrazoxane from cycle 1 resulted in a 85% reduction in the risk of cardiotoxicity (hazard ratio: 0.15; 95% CI: 0.02–0.99) compared to those who did not receive dexrazoxane [23]. This indicates that the timing of dexrazoxane may have significantly affected the risk of cardiotoxicity.

Both neutropenic fever, although not statistically significant and the use of dexrazoxane were observed more frequently at Herlev. Myelosuppression may be exacerbated when dexrazoxane is administered in combination with doxorubicin [25, 26], and we cannot exclude the possibility that dexrazoxane may have contributed to the higher incidence of neutropenic fever observed at Herlev. It did not result in any toxicity-related deaths; however, it may have negatively affected OS by delaying subsequent treatment options. On the contrary, two other studies including patients with both STS and breast cancer did not report an increased incidence of neutropenia when anthracycline‑based treatment was combined with dexrazoxane [27, 28].

Dexrazoxane has previously been shown to reduce the risk of anthracycline-associated cardiotoxicity [29, 30]. One meta-analysis reported a statistically significant benefit in favor of dexrazoxane for the occurrence of heart failure (Relative Risk [RR]: 0.29, 95% CI: 0.20–0.41) [31] and a smaller retrospective study reported a 7% incidence of cardiotoxicity in patients receiving cumulative anthracycline doses of up to 750 mg/m^2^ [32]. In addition, a meta-analysis in patients with breast cancer reported that dexrazoxane reduced the risk of clinical heart failure (RR: 0.19, 95% CI: 0.09–0.40; *p* < 0.001) and cardiac events (RR: 0.36, 95% CI: 0.27–0.49; *p* < 0.001) irrespective of previous exposure to anthracyclines. The response rate, OS, and progression-free survival were not affected by dexrazoxane [33].

Despite the Food and Drug Administration (FDA) approval and its well described cardio-protective effects, dexrazoxane is not widely incorporated into routine clinical practice. This is largely due to concerns regarding potential attenuation of chemotherapeutic efficacy [29], although it has been reported that the use of dexrazoxane is unlikely to affect anthracycline efficacy [26, 34, 35]. Other contributing factors include concerns about secondary malignancies, increased risk of myelosuppression [26, 36] and finally the cost of dexrazoxane. At present, the 2022 ESC cardio-oncology guideline does not recommend the routine use of dexrazoxane in patients undergoing anthracycline-based treatment. Dexrazoxane should only be considered for adult patients with cancer who are at high risk of cardiotoxicity [5]. The 2016 American Society of Clinical Oncology clinical practice guideline recommend consideration of a cardio-protective strategy such as dexrazoxane, liposomal formulations, or continuous infusion in patients who will receive high-dose anthracyclines (≥250 mg/m^2^ doxorubicin) [37]. Pegylated liposomal doxorubicin has previously shown efficacy in STS with a lower toxicity profile, however studies are limited [38]. In addition, a recently published viewpoint has suggested that dexrazoxane should be considered for patients at high risk who are receiving cumulative doxorubicin doses of ≥250 mg/m^2^ [39].

In general, it’s challenging to compare the incidence of cardiotoxicity in retrospective studies. The incidence can vary substantially largely due to inconsistencies across studies in the definitions of cardiotoxicity, the approaches to cardiac monitoring, the characteristics of the populations studied, the specific anthracycline regimens administered, the administration of dexrazoxane, methodological differences in study design and follow-up periods, and finally the concomitant use of radiation therapy [17].

## Strengths and limitations

To our knowledge, there are limited reports on the incidence of cardiotoxicity in patients with STS receiving doxorubicin in combination with dexrazoxane. The FDA approval of dexrazoxane was based primarily on studies in patients with breast cancer. This study therefore expands the existing evidence base. Furthermore, this is a population‑based study and all Danish patients with STS treated with doxorubicin and olaratumab were included, providing real‑world data that reflect routine clinical practice. Nevertheless, only 35 patients were included from Aarhus and the small sample size limits the ability to draw meaningful comparisons between the two treatment centers. In addition, a large proportion of patients had only one or no cardiac imaging performed introducing a risk of underdetecting cardiotoxicity. Moreover, only seven patients developed cardiotoxicity, and the limited number of events precludes any definitive conclusions. Since it was a retrospective study no power calculations were made. A larger retrospective study is currently investigating the incidence of cardiotoxicity, OS and dexrazoxane-associated toxicities in a Danish population‑based cohort of patients with STS. The forthcoming results may provide a more detailed understanding of the cardio-protective properties of dexrazoxane and help to clarify concerns surrounding its use. Ultimately, a larger prospective study is warranted before any definitive recommendations can be made.

In conclusion doxorubicin and olaratumab with dexrazoxane were associated with a low incidence of cardiotoxicity and OS consistent with previously published data.

## Data Availability

The data generated and analyzed in this study are stored in a secure REDCap database at our institution. Because the dataset contains personally identifiable information, including Danish Civil Registration Numbers (CPR), the data cannot be made open. Access to the data may be granted upon reasonable request and contingent on approval from the institutional data protection authorities and compliance with applicable data protection regulations.

## References

[1] Burningham Z, Hashibe M, Spector L, Schiffman JD. The epidemiology of sarcoma. Clin Sarcoma Res. 2012;2:14. 10.1186/2045-3329-2-14 23036164 PMC3564705

[2] Casali PG, Abecassis N, Bauer S, Biagini R, Bielack S, Bonvalot S, et al. Soft tissue and visceral sarcomas: ESMO–EURACAN Clinical Practice Guidelines for diagnosis, treatment and follow-up. Ann Oncol. 2018;29:51–67. 10.1093/annonc/mdy096 29846498

[3] Simůnek T, Stérba M, Popelová O, Adamcová M, Hrdina R, Gersl V. Anthracycline-induced cardiotoxicity: overview of studies examining the roles of oxidative stress and free cellular iron. Pharmacol Rep. 2009;61:154–71. 10.1016/s1734-1140(09)70018-0 19307704

[4] Dempke WCM, Zielinski R, Winkler C, Silberman S, Reuther S, Priebe W. Anthracycline-induced cardiotoxicity – are we about to clear this hurdle? Eur J Cancer. 2023;185:94–104. 10.1016/j.ejca.2023.02.019 36966697

[5] Lyon AR, López-Fernández T, Couch LS, Asteggiano R, Aznar MC, Bergler-Klein J, et al. 2022 ESC Guidelines on cardio-oncology developed in collaboration with the European Hematology Association (EHA), the European Society for Therapeutic Radiology and Oncology (ESTRO) and the International Cardio-Oncology Society (IC-OS). Eur Heart J. 2022;43:4229–361. 10.1093/eurheartj/ehac244 36017568

[6] Shapira J, Gotfried M, Lishner M, Ravid M. Reduced cardiotoxicity of doxorubicin by a 6-hour infusion regimen. A prospective randomized evaluation. Cancer. 1990;65:870–3. 10.1002/1097-0142(19900215)65:4<870::aid-cncr2820650407>3.0.co;2-d 2297656

[7] Vejpongsa P, Yeh ET. Prevention of anthracycline-induced cardiotoxicity: challenges and opportunities. J Am Coll Cardiol. 2014;64: 938–45. 10.1016/j.jacc.2014.06.1167 25169180

[8] Tap WD, Jones RL, Van Tine BA, Chmielowski B, Elias AD, Adkins D, et al. Olaratumab and doxorubicin versus doxorubicin alone for treatment of soft-tissue sarcoma: an open-label phase 1b and randomised phase 2 trial. Lancet. 2016;388:488–97. 10.1016/S0140-6736(16)30587-6 27291997 PMC5647653

[9] Tap WD WA, Schöffski P, Martin-Broto J, Krarup-Hansen A, Ganjoo KN, Yen CC, et al. Effect of doxorubicin plus olaratumab vs doxorubicin plus placebo on survival in patients with advanced soft tissue sarcomas: the ANNOUNCE randomized clinical trial. JAMA. 2020;323:1266–76. 10.1001/jama.2020.1707 32259228 PMC7139275

[10] Judson I, Verweij J, Gelderblom H, Hartmann JT, Schöffski P, Blay JY, et al. Doxorubicin alone versus intensified doxorubicin plus ifosfamide for first-line treatment of advanced or metastatic soft-tissue sarcoma: a randomised controlled phase 3 trial. Lancet Oncol. 2014;15:415–23. 10.1016/S1470-2045(14)70063-4 24618336

[11] Seeber LW A, Romeder F, Szkandera J, Kuehr T, Kostner S, Pichler, et al. Olaratumab plus anthracyline in advanced/metastatic soft tissue sarcoma: data of real-world utilization in Austria. Wien Klin Wochenschr. 2021;133:21–5. 10.1007/s00508-019-01556-1 31620878

[12] Striefler JK, Brandes F, Baur A, Pfitzner BM, Kaul D, Rau D, et al. Combination therapy with Olaratumab/doxorubicin in advanced or metastatic soft tissue sarcoma – a single-Centre experience. BMC Cancer. 2020;20:68. 10.1186/s12885-020-6551-y 31996176 PMC6988236

[13] Jørgensen PH, Lausten GS, Pedersen AB. The Danish Sarcoma Database. Clin Epidemiol. 2016;8:685–90. 10.2147/CLEP.S99495 27822116 PMC5094641

[14] Trojani M, Contesso G, Coindre JM, Rouesse J, Bui NB, de Mascarel A, et al. Soft-tissue sarcomas of adults; study of pathological prognostic variables and definition of a histopathological grading system. Int J Cancer. 1984;33:37–42. 10.1002/ijc.2910330108 6693192

[15] Freites-Martinez A, Santana N, Arias-Santiago S, Viera A. Using the common terminology criteria for adverse events (CTCAE – Version 5.0) to evaluate the severity of adverse events of anticancer therapies. Actas Dermosifiliogr (Engl Ed). 2021;112:90–2. 10.1016/j.ad.2019.05.009 32891586

[16] Khouri MG, Douglas PS, Mackey JR, Martin M, Scott JM, Scherrer-Crosbie M, et al. Cancer therapy-induced cardiac toxicity in early breast cancer: addressing the unresolved issues. Circulation. 2012;126:2749–63. 10.1161/CIRCULATIONAHA.112.100560 23212997 PMC3667651

[17] Vitfell-Rasmussen J, Krarup-Hansen A, Vaage-Nilsen M, Kümler T, Zerahn B. Real-life incidence of cardiotoxicity and associated risk factors in sarcoma patients receiving doxorubicin. Acta Oncol. 2022;61:801–8. 10.1080/0284186X.2022.2082884 35695121

[18] Kamphuis JAM, Linschoten M, Cramer MJ, Doevendans PA, Asselbergs FW, Teske AJ. Early- and late anthracycline-induced cardiac dysfunction: echocardiographic characterization and response to heart failure therapy. Cardiooncology. 2020;6:23. 10.1186/s40959-020-00079-3 33072403 PMC7557080

[19] Jones RL, Wagner AJ, Kawai A, Tamura K, Shahir A, Van Tine BA, et al. Prospective evaluation of doxorubicin cardiotoxicity in patients with advanced soft-tissue sarcoma treated in the ANNOUNCE phase III randomized trial. Clin Cancer Res. 2021;27:3861–6. 10.1158/1078-0432.CCR-20-4592 33632930 PMC8282740

[20] Cardinale D, Colombo A, Bacchiani G, Tedeschi I, Meroni CA, Veglia F, et al. Early detection of anthracycline cardiotoxicity and improvement with heart failure therapy. Circulation. 2015;131:1981–8. 10.1161/CIRCULATIONAHA.114.013777 25948538

[21] Larsson JE, Nielsen M, Zerahn B. Incidence of cardiotoxicity assessed with CZT-ERNA in patients undergoing contemporary cancer therapy: a 12-year real-world experience. Cardiooncology. 2026;12:40. 10.1186/s40959-026-00457-3 41703591 PMC13014980

[22] Lotrionte M, Biondi-Zoccai G, Abbate A, Lanzetta G, D’Ascenzo F, Malavasi V, et al. Review and meta-analysis of incidence and clinical predictors of anthracycline cardiotoxicity. Am J Cardiol. 2013;112:1980–4. 10.1016/j.amjcard.2013.08.026 24075281

[23] Jang KH, Wallins JS, Lin IH, Movva S, Tap WD, Liu JE, et al. Association of early dexrazoxane with reduced cardiotoxicity risk in sarcoma patients treated with anthracycline chemotherapy. Oncologist. 2025;30:370. 10.1093/oncolo/oyaf370. PMC12707907 41206057

[24] Camilli M, Cipolla CM, Dent S, Minotti G, Cardinale DM. Anthracycline cardiotoxicity in adult cancer patients: JACC: CardioOncology State-of-the-Art Review. JACC CardioOncol. 2024;6:655–77. 10.1016/j.jaccao.2024.07.016 39479333 PMC11520218

[25] Pfizer Inc. Zinecard (dexrazoxane) for injection: prescribing information. Silver Spring, MD: US Food and Drug Administration; 2014. [cited 2026 February]. Available from: https://www.accessdata.fda.gov/drugsatfda_docs/label/2014/020212s017lbl.pdf

[26] de Baat EC, Mulder RL, Armenian S, Feijen EA, Grotenhuis H, Hudson MM, et al. Dexrazoxane for preventing or reducing cardiotoxicity in adults and children with cancer receiving anthracyclines. Cochrane Database Syst Rev. 2022;9:CD014638. 10.1002/14651858.CD014638.pub2. 36162822 PMC9512638

[27] Lopez M, Vici P, Di Lauro K, Conti F, Paoletti G, Ferraironi A, et al. Randomized prospective clinical trial of high-dose epirubicin and dexrazoxane in patients with advanced breast cancer and soft tissue sarcomas. J Clin Oncol. 1998;16:86–92. 10.1200/JCO.1998.16.1.86 9440727

[28] Marty M, Espié M, Llombart A, Monnier A, Rapoport BL, Stahalova V. Multicenter randomized phase III study of the cardioprotective effect of dexrazoxane (Cardioxane) in advanced/metastatic breast cancer patients treated with anthracycline-based chemotherapy. Ann Oncol. 2006;17:614–22. 10.1093/annonc/mdj134 16423847

[29] Swain SM, Whaley FS, Gerber MC, Weisberg S, York M, Spicer D, et al. Cardioprotection with dexrazoxane for doxorubicin-containing therapy in advanced breast cancer. J Clin Oncol. 1997;15:1318–32. 10.1200/JCO.1997.15.4.1318 9193323

[30] Liesse K, Harris J, Chan M, Schmidt ML, Chiu B. Dexrazoxane significantly reduces anthracycline-induced cardiotoxicity in pediatric solid tumor patients: a systematic review. J Pediatr Hematol Oncol. 2018;40:417–25. 10.1097/MPH.0000000000001118 29432315 PMC6059999

[31] van Dalen EC, Caron HN, Dickinson HO, Kremer LC. Cardioprotective interventions for cancer patients receiving anthracyclines. Cochrane Database Syst Rev. 2008;16:CD003917. 10.1002/14651858.CD003917.pub3. 18425895

[32] Schuler MK, Gerdes S, West A, Richter S, Busemann C, Hentschel L, et al. Efficacy and safety of Dexrazoxane (DRZ) in sarcoma patients receiving high cumulative doses of anthracycline therapy – a retrospective study including 32 patients. BMC Cancer. 2016;16:619. 10.1186/s12885-016-2654-x 27507014 PMC4977890

[33] Macedo AVS, Hajjar LA, Lyon AR, Nascimento BR, Putzu A, Rossi L, et al. Efficacy of dexrazoxane in preventing anthracycline cardiotoxicity in breast cancer. JACC CardioOncol. 2019;1:68–79. 10.1016/j.jaccao.2019.08.003 34396164 PMC8352186

[34] Schwartz CL, Wexler LH, Krailo MD, Teot LA, Devidas M, Steinherz LJ, et al. Intensified chemotherapy with dexrazoxane cardioprotection in newly diagnosed nonmetastatic osteosarcoma: a report from the Children’s Oncology Group. Pediatr Blood Cancer. 2016;63:54–61. 10.1002/pbc.25753 26398490 PMC4779061

[35] Tian Z, Yang Y, Yang Y, Zhang F, Li P, Wang J, et al. High cumulative doxorubicin dose for advanced soft tissue sarcoma. BMC Cancer. 2020;20:1139. 10.1186/s12885-020-07663-x 33228579 PMC7684756

[36] Upshaw JN, Parson SK, Buchsbaum RJ, Schlam I, Ruddy KJ, Durani U, et al. Dexrazoxane to prevent cardiotoxicity in adults treated with anthracyclines: JACC: CardioOncology Controversies in Cardio-Oncology. JACC CardioOncol. 2024;6:322–4. 10.1016/j.jaccao.2024.02.004 38773999 PMC11103024

[37] Armenian SH, Lacchetti C, Lenihan D. Prevention and monitoring of cardiac dysfunction in survivors of adult cancers: American Society of Clinical Oncology Clinical Practice Guideline Summary. J Oncol Pract. 2017;13:270–5. 10.1200/JOP.2016.018770 27922796

[38] Pham T, Krogh HR, Rossen P, Aggerholm-Pedersen N. The use of pegylated liposomal doxorubicin in metastatic soft tissue sarcoma. Acta Oncol. 2025;64:558–63. 10.2340/1651-226X.2025.43263 40269485 PMC12041797

[39] Zheng H, Zhan H. Preventing anthracycline-associated heart failure: what is the role of dexrazoxane?: JACC: CardioOncology Controversies in Cardio-Oncology. JACC CardioOncol. 2024;6:318–21. 10.1016/j.jaccao.2024.01.004 38774009 PMC11103021

